# Mimicking p14ARF Phosphorylation Influences Its Ability to Restrain Cell Proliferation

**DOI:** 10.1371/journal.pone.0053631

**Published:** 2013-01-07

**Authors:** Maria Vivo, Michela Ranieri, Federica Sansone, Cristina Santoriello, Raffaele A. Calogero, Viola Calabrò, Alessandra Pollice, Girolama La Mantia

**Affiliations:** 1 Department of Structural and Functional Biology, University of Naples “Federico II”, Naples, Italy; 2 Harvard Department of Stem Cell and Regenerative Biology, Harvard University, Cambridge, Massachusetts, United States of America; 3 Department of Computer Science, University of Turin, Turin, Italy; South China Sun Yat-sen University Cancer Center, China

## Abstract

The INK4a/ARF locus on the short arm of chromosome 9 is one of the most frequently altered loci in human cancer. It is generally accepted that ARF is involved in oncogenic checkpoint pathways by sensitizing incipient cancer cells to undergo growth arrest or apoptosis through both p53-dependent and independent pathways. While intensive studies have been focused on ARF activation at the transcriptional level, only recently mechanisms governing ARF turnover have been identified. Here, we show for the first time that p14ARF is a PKC target. Prediction analysis showed many potential phosphorylation sites in PKC consensus sequences within ARF protein, and, among them, the threonine at position 8 was the most conserved. Substitution of this threonine influences both ARF stability and localization. Furthermore, a phosphomimetic ARF mutation reduces the ability to arrest cell growth although the ability to bind MDM2 and stabilize p53 result unaffected. Thus we propose that phosphorylation of ARF in both immortalized and tumor cell lines could be a mechanism to escape ARF surveillance following proliferative and oncogenic stress.

## Introduction

The ARF protein is encoded by the alternative reading frame of the INK4a locus, one of the most frequent sites of genetic loss in human cancers. Over the last years, a direct contribution of ARF to tumor formation has been documented using genetic analysis of tumors, molecular and cell biology methods and animal models [1], [2], [3]. Both the human (p14ARF) and mouse (p19ARF) proteins function as important sensors of hyper-proliferative stimuli acting to restrict cell proliferation through both p53-dependent and independent pathways [4], [5]. Under normal conditions the p53 protein is kept at low levels by its rapid turn over, and exists in a latent and inactive form. Activation of p53 in response to cellular stresses is mediated, at least in part, by inhibition of MDM2 functions [6]. In normal conditions, primary cells contain low levels of ARF. However, when stimulated to proliferate by oncogenic proteins such as Myc, E2F, E1A, oncogenic Ras and v-Abl [7], [8], [9], [10], ARF levels increase. This phenomenon is generally accompanied by a parallel disruption of the inhibitory interaction between Mdm2 and p53, resulting in the accumulation of transcriptionally active p53 that induces specific responses such as cell-cycle arrest or apoptosis [11], [12]. However, genetic analysis of tumors and the evidence that ARF can induce cell-cycle arrest in cells lacking Mdm2 and p53 support the notion that ARF might act independently of MDM2 and p53 [2], [5], [13], [14], [15]. Furthermore, the discovery of a plethora of ARF interactors and the observation that also viral, genotoxic, hypoxic and oxidative stresses activate an ARF-dependent response, suggest that ARF has a wider role to protect the cell [16]. Given its strong ability to block growth and proliferation, cells must develop mechanisms that promptly reduce either its expression or functions when its activity is no longer required. While ARF activation, mostly occurring through transcriptional activation, has been the focus of intensive studies [17], [18], [19], [20], [21] only recently the mechanisms regulating ARF protein turnover started to be elucidated. ARF degradation can be regulated by the proteasome by both ubiquitin independent [22] or lysine-independent N-terminal ubiquitylation mechanisms [23]. Recently, a specific ARF ubiquitin ligase, ULF has been identified [23], [24]. Moreover, it has been observed that in primary cells ARF turnover is very fast: these cells express very low levels of protein, which could be promptly stabilized upon proteasome inhibitor treatment. Conversely, ARF protein appears to be very stable in cancer cells where proteasome-mediated ARF degradation is severely impaired [24].

It has been reported that Protein Kinase C alpha (PKCα) is involved in the stabilization of exogenously expressed ARF protein levels [25]. Multiple PKC isoforms have been associated with epidermal keratinocyte differentiation program and some of them are activated by calcium [26]. We have previously shown that both p14ARF transcript and protein levels increase upon calcium-induced keratinocyte differentiation. Here we present data showing that endogenous human ARF protein levels increase following stimuli known to activate the PKC pathway in both tumor and immortilized keratinocyte cell lines. A careful examination of ARF protein sequence revealed three potential PKC phosphorylation sites. Among them, Threonine at position 8 lies within the most conserved region encompassing amino acids 2–14 and is required for the activity and nuclear localization of ARF protein [26], [27], [28]. Our data indicate that mutation of this residue, mimicking a phosphorylation status of the protein, influences both ARF activity and cellular localization.

## Results

### p14ARF Protein Levels Increase following Calcium and TPA Treatment

**Figure 1:**
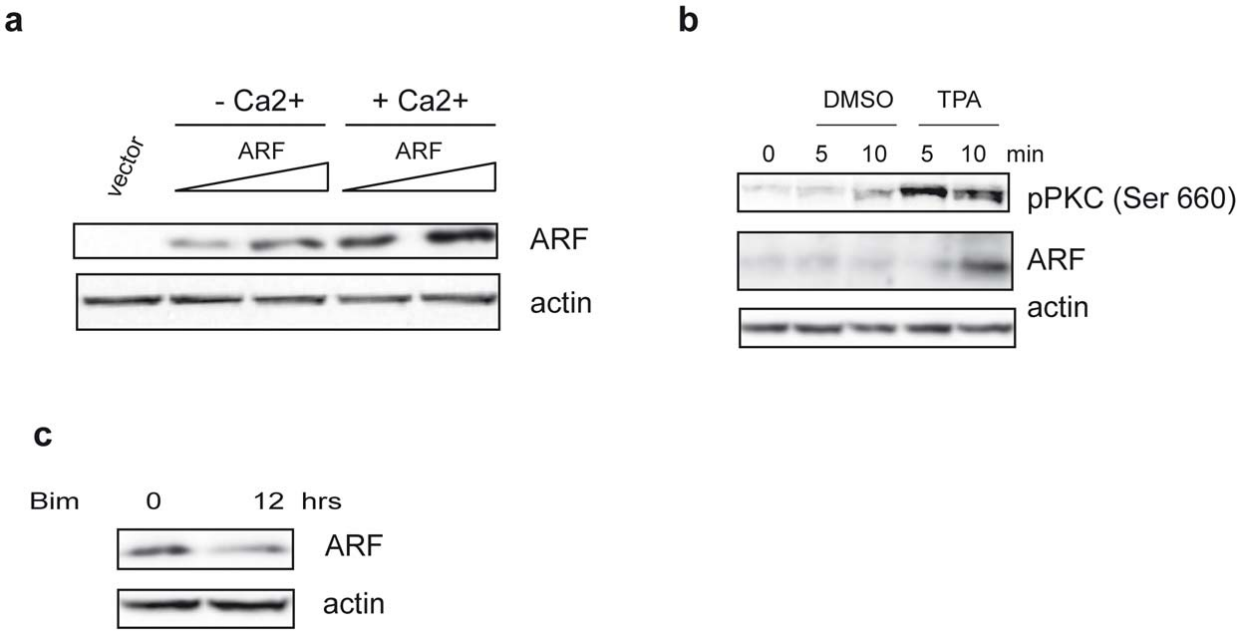
SDS-PAGE analysis of ARF protein levels. a) HaCaT cells were transfected with an empty plasmid (vector) and increasing amount of p14ARF expression plasmid. Twenty-four hours after transfection cells were treated or not with 2mM Ca^2+^ as indicated. Cellular extracts were immunoblotted and analysed with anti His antibody to detect exogenous ARF levels and anti actin as loading control. b) HaCaT cells were treated with 10µM TPA for different time points. Protein extracts were subjected to WB with anti ARF (C-18) and an anti pPKC pan antibody that recognizes all the PKC isoforms phosphorylated at a carboxy-terminal residue homologous to serine 660 of PKC β II (activated pPKC). Actin was used as loading control. c) HaCaT cells were treated with 5µM Bisindolylmaleimide (Bim) for 12 hours (hrs) and analysed with anti ARF and anti actin antibodies.

We had previously shown that, during calcium induced differentiation of transformed human keratinocyte cells (HaCaT), p14ARF increases both at protein and transcriptional levels [29]. To understand if the increase in ARF levels was due to a transcription independent mechanism, HaCaT cells were transfected with increasing amounts of a plasmid expressing p14ARF and treated with 2mM calcium for 12h in presence of serum. Western blot analysis of total protein extracts (Fig. 1a) shows that ARF levels increase following stimulation. Quantification of multiple esperiments shows a two fold increase of ARF protein levels following calcium treatment (2±0,5). It has been reported that PKCα, which can be activated by Ca^2+^, is involved in TPA-mediated stabilization of p14ARF [25]. Thus we analysed ARF protein levels in HaCaT cells treated with 10µM TPA (Fig. 1b). The experiment shows that TPA induces a fast PKC activation, followed by an increase of ARF protein levels after 10 minutes of treatment (2.5±0,09). Accordingly, a decrease of almost 70% ARF levels (0,3±0,05) was obtained when cells were grown in presence of 5µM bisindolylmaleimide, a known inhibitor of PKC activation (Fig. 1c). Similar results were obtained using the p53 deficient human lung cancer cell line H1299 expressing endogenous ARF (data not shown). Altogether, these experiments prompted us to further investigate on the potential role of PKC and phosphorylation on p14ARF regulation.

### p14ARF Interacts with and is Phosphorylated by PKC in vitro

**Figure 2:**
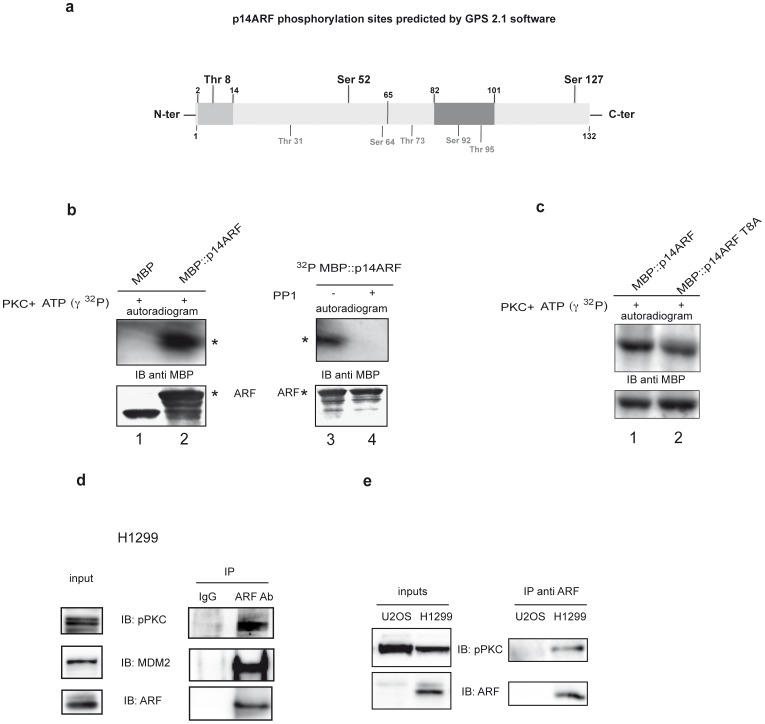
ARF is a substrate of PKC and PP1 in vitro. a) Diagramatic scheme of p14ARF phosphorylation sites identified in silico. In light gray is indicated the conserved 2–14 aminoacid stretch within the N-terminal exon 1β encoded domain (aminoacid 1–65), while in dark gray the exon 2 nuclear localization signal. PKC phosphorylation sites are shown in bold above the scheme, while other kinases target sites are denoted in gray below the scheme b) In vitro phosphorylation assay. MBP (lane 1) and MBP::ARF (lane 2) were incubated with labelled γATP and catalytic PKC subunit. Reactions were then subjected to SDS-page and WB followed by ON exposure (upper panel). The lower panel shows the same filter immunoblotted with anti MBP antibodies. Detections of MBP::ARF is denoted by (*) on the autoradiograms and WBs. Purified phosphorylated MBP::ARF was divided in two aliquots and incubated (lane 4) or not (lane 3) with catalytic PP1 and processed as described in a). c) In vitro phosphorylation assay of MBP::T8A mutant. d) Co-immunoprecipitation of pPKC with ARF: H1299 lysates were incubated with anti-ARF antibody or with mouse IgG as negative control. Samples were analyzed by immunoblotting with anti pPKC Ser660, anti ARF and anti MDM2 antibodies.

Analysis of ARF protein sequence with GPS 2.1 [30] software revealed the presence of three potential PKC phosphorylation sites. Among these, Threonine 8 and Serine 52 lie in the functional active domain of the protein, while Serine 127 in the C-ter region of the protein (Fig. 2a). Interestingly, threonine 8 is conserved among all ARF orthologs. We thus performed an in vitro assay to test ARF ability to be phosphorylated by PKC. Phosphorylation assay was performed using a purified MBP::ARF protein and a commercially available Protein Kinase C phosphorylation assay. A reaction containing only the maltose binding protein was performed in parallel, as negative control. Autoradiography showed a clean phosphorylated MBP-ARF signal, indicating that ARF is actually a PKC substrate (Fig. 2b, compare lane 2 with lane 1). A fusion protein in which the three PKC consensus sequences were simultaneously mutated was used as control. This mutant was not phosphorylated in these experimental conditions, thus confirming the specificity of the kinase assay (data not shown). As many PKC substrates are dephosphorylated by PP1 [31], equal aliquots of the reaction, after kinase incubation, were subjected or not to a dephosphorylation assay using the Protein Phosphatase-1 (PP1) catalytic subunit. As shown in Fig2, panel B, the phosphorylated ARF signal disappeared upon PP1 treatment (compare lane 4 with lane 3). The ability of PKC to phosphorylate a p14ARF mutant where the threonine 8 was replaced with an alanine (MBP::p14ARF T8A) was also analysed. As shown in Fig. 2c this mutant is still a PKC substrate even if at lesser extent than the wt protein.

We next verified the physical association between ARF and PKC by coimmunoprecipitation experiment in H1299 cells expressing endogenous p14ARF (Fig. 2d). As a negative control we used the human osteosarcoma U2OS cell line that is wild type for p53 but lacks expression of p16 and p14ARF proteins. Total extracts were immunoprecipitated with anti ARF antibody and complexes probed with anti ARF and anti phospho-PKC pan antibody. Protein complexes were also assayed with an antibody recognizing a well known ARF protein partner, the MDM2 oncoprotein, as positive control. As shown in Fig. 2 (panel d and e), a complex between ARF and activated PKC is exclusively detected in H1299 cells.

### Mutation of Threonine 8 does not Affect ARF Folding

**Figure 3:**
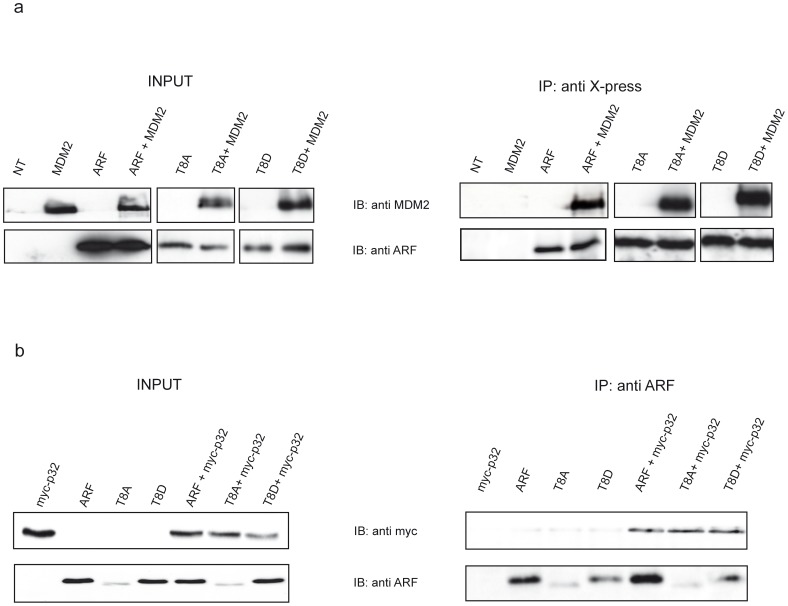
Mutation of Threonine 8 does not affect ARF folding. a) U2OS cells were transfected with plasmids encoding X-press tagged version of either wild type or mutants ARF proteins alone or in combination with plasmid encoding human MDM2 protein. Cellular lysates were immunoprecipitated with anti X-press antibody and samples analyzed by immunoblotting with anti MDM2 and ARF antibodies. b) cells were trasfected with myc tagged p32 encoding plasmid and wt and mutants ARF as described in a). Cellular lysates were immunoprecipitated with anti ARF and analysed by IB with anti myc and anti ARF antibodies.

The conserved threonine at position 8 lies in a twelve amino acid stretch (region 2–14, see Fig. 2a) strictly required for ARF biological activity. To analyse the role of this residue in ARF functions, we constructed ARF mutants where threonine 8 was replaced either by an alanine residue, that cannot be phosphorylated (T8A mutant), or by an aspartic acid (T8D mutant) that mimics the phosphorylated status of the protein. To rule out the possibility that the inserted mutations could have resulted in an uncorrectly folded protein, we tested the binding to MDM2, interacting with the N-terminal ARF region [28], and to p32 that binds ARF through the C-terminal domain [32]. U2OS cells were transfected with expression plasmids encoding human MDM2 or myc tagged p32, and/or X-press tagged wild type or mutant ARF proteins (Fig. 3). Immunoprecipitated complexes were blotted and probed with anti-MDM2, anti- myc and anti-ARF antibodies. The experiments show that the substitution of threonine at position 8 does not affect ARF binding to MDM2 and p32 (Fig. 3a and b).

### Mutation of Threonine 8 Affects ARF Protein Turn-over

**Figure 4:**
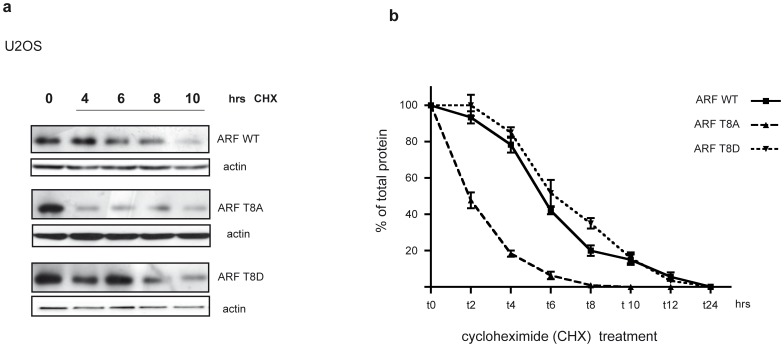
Protein turn-over analysis. a) Half-life analysis of the wt and mutated p14ARF in human U2OS cells. Cells were transfected with p14ARF wt, T8A and T8D plasmids and treated with cycloheximide (CHX) for the indicated times two days after transfection. ARF and actin levels were analysed by WB of total extracts. b) The plot represents half-life analysis of wt and mutant ARFs. Band intensities were quantified by Image J analysis and actin normalized before being plotted in graph. The amount of protein at different time points is expressed as percentage of total protein, i.e. protein amount at t0. Each profile represents the mean of three independent transfections and WB experiments. Standard deviations are also shown.

Immunoblots of total cell lysates reproducibly showed lower levels of T8A mutant compared to wt and T8D mutant suggesting lower protein stability. To explore this possibility, half-life studies of the wt and mutant proteins were performed using the protein synthesis inhibitor cycloheximide (Fig. 4). Half-life analyses showed that reduced expression of T8A mutant correlated with decreased stability. Wild-type ARF showed a half-life of 6 hours, as previously reported [33]. While T8D mutant stability is comparable to that of the wt, the half-life of the T8A mutant is reduced to 2 hours, indicating that this residue could be involved in p14ARF stability (Fig. 4a and 4b).

### Mimicking Thr8 Phosphorylation Induces ARF Accumulation in the Cytoplasm and Nucleus

It has been extensively shown that ARF amino acid stretch 2–14 is not only involved in MDM2 binding and ARF mediated cell cycle arrest but also contribute to p14ARF nucleolar compartimentalization [28], [33]. Thus we analysed the effect of T8 mutations on ARF subcellular localization following transfections in U2OS cells.

**Figure 5:**
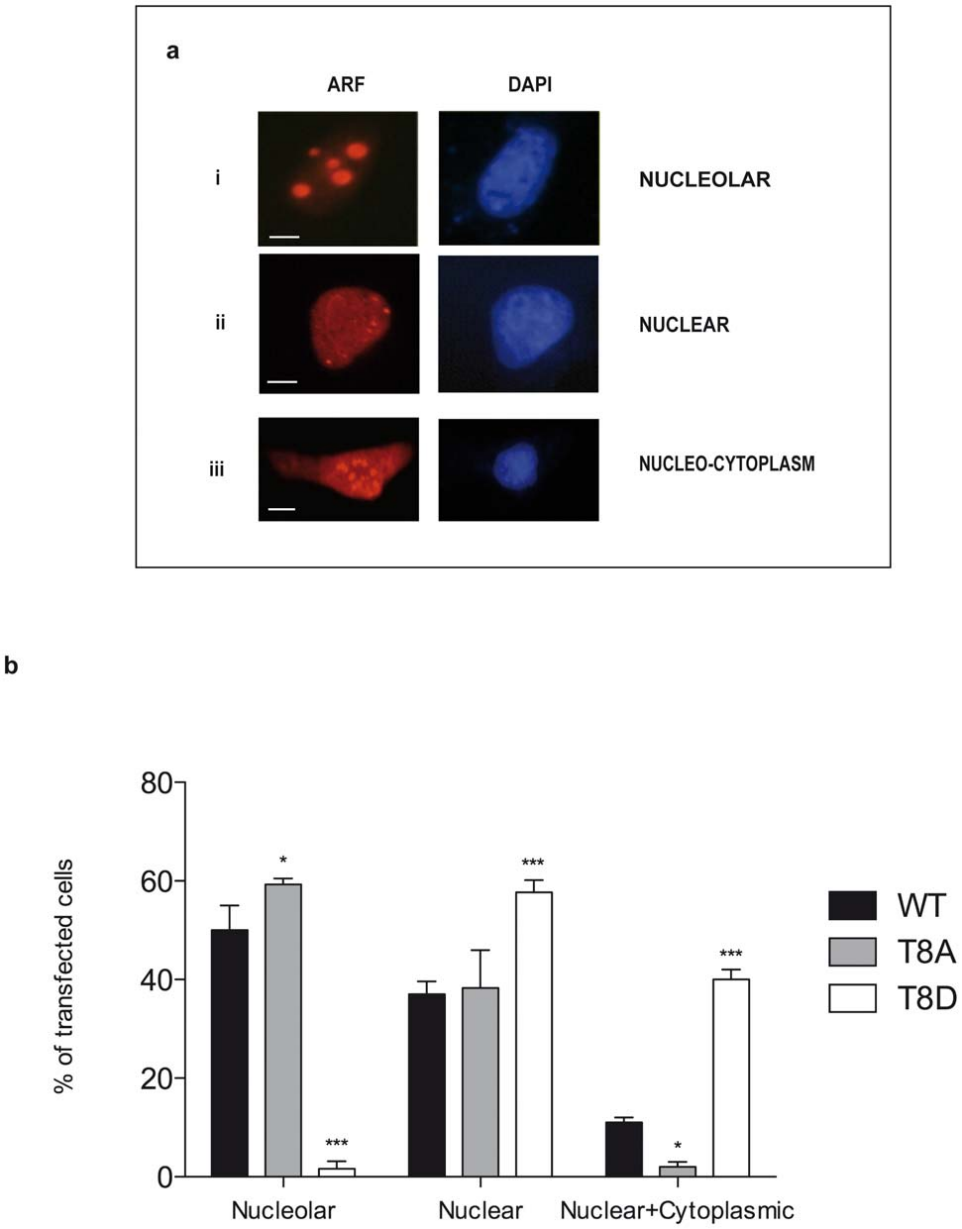
ARF localization analysis. U2OS cells were transfected with p14ARF expression plasmids (wt or mutants) and visualized by IF with anti histidine antibody. Nuclei were stained with DAPI. Images were taken with a Nikon fluorescent microscope. a) Visualization of the three different wt ARF localization patterns are indicated with i, ii and iii. Scale bar: i, ii = 3µm; iii = 7µm. b) The histogram, representing the mean of three independent experiments, reports the percentage of transfected cells showing each localization pattern. Standard deviations are also shown. Asterisks indicate statistically significant differences between the sample and wt ARF (* = p<0.05; *** = p<0.001).

As expected, following transfection wt ARF protein presents different localization patterns with cells showing three different stainings: nucleolar (Fig. 5a panel i), nuclear (Fig. 5a panel ii) and nucleo-cytoplasmic (Fig. 5a panel iii). Quantitation of this result showed, for the wt protein, a clear nucleolar and nuclear distribution pattern in the majority of transfected cells (90%), and a nuclear-cytoplasmic distribution in the remaining 10% of cells (Fig. 5b). The T to A substitution did not significantly compromise the number of cells showing a nuclear localization as shown by quantitation in Fig. 5b. However, replacement of the same residue with an aspartic acid dramatically reduced the proportion of cells showing exclusively nucleolar ARF localization and led to an increase in the percentage of cells with nuclear and nuclear-cytoplasmic ARF staining (Fig. 5b). Experiments performed with GFP fusion proteins gave similar results (data not shown). These data suggest that a mutation mimicking T8 phosphorylation can induce accumulation of the protein both in the cytoplasm and in the nucleus.

### Cytoplasmic Localized ARF Protein is Phosphorylated

**Figure 6:**
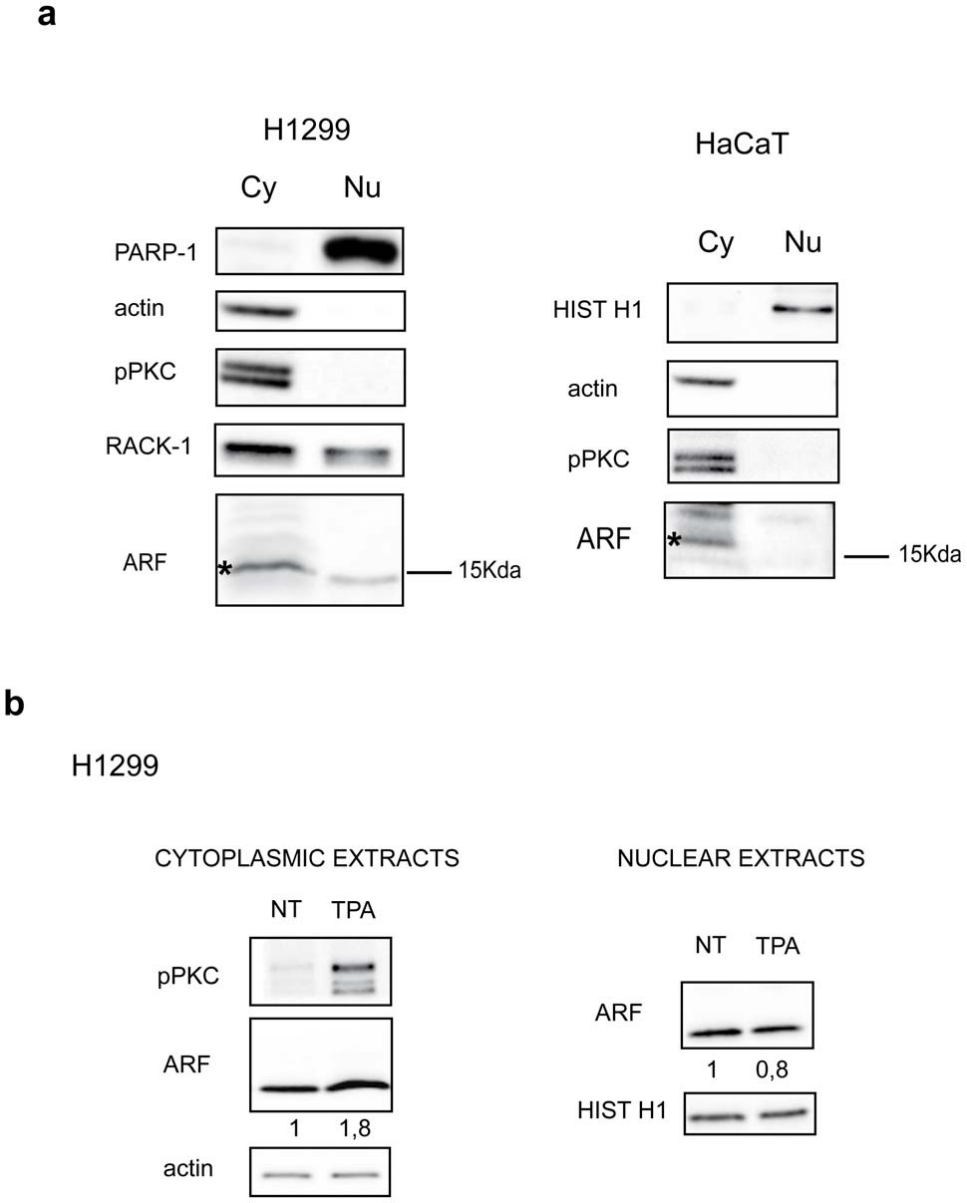
Sub-cellular fractionaction. H1299 cells, endogenously expressing p14ARF, were subjected to subcellular fractionation. a) Left panel: cytoplasmic (Cy) and nuclear extracts (Nu) from equal number of cells were subjected to SDS-page and immunoblot with anti PARP-1 (nuclear), actin and RACK-1 antibodies (cytoplasm), anti pPKC and anti ARF antibodies. Right panel: HaCaT cells were subjected to subcellular fractionation and analysed as previously described. Histone H1 is a control of nuclear extracts. Cytoplasmic ARF species are indicated by an asterisk (*). b) H1299 cells were treated with TPA and subsequently subjected to subcellular fractionation. Equal amount of cytoplasmic (left panel) or nuclear (right panel) extracts were immunoblotted with anti ARF, pPKC, and actin and histone H1 as loading control for cytoplasm and nucleus respectively, as described. WB shown are representative of at least three independent experiments. Normalized ARF band intensities, shown below each corresponding band, are expressed as fold enrichment respect to untreated samples arbitrarily set to 1 (see material and methods for details).

Endogenous ARF localization was then analysed in tumor cells such as H1299 by western blot of cytoplasmic and nuclear extracts. Efficient fractionation was checked with anti PARP-1 for nucleus and anti actin or RACK-1 antibodies for the cytoplasm as these proteins are mainly enriched in these cellular compartments (Fig. 6a and [34]). Using different ARF antibodies, we consistently found an ARF immunoreactive band both in nuclear and cytoplasmic extracts (Fig. 6a and data not shown). Moreover, fractionation experiments, performed in the HaCaT cell line, showed ARF expression almost exclusively in the cytoplasm (Fig. 6a right panel), in agreement with our previously published immunofluorescence data [29]. Western blot analysis also shows that activated PKC is mainly localized in the cytosol (Fig. 6a).

**Figure 7:**
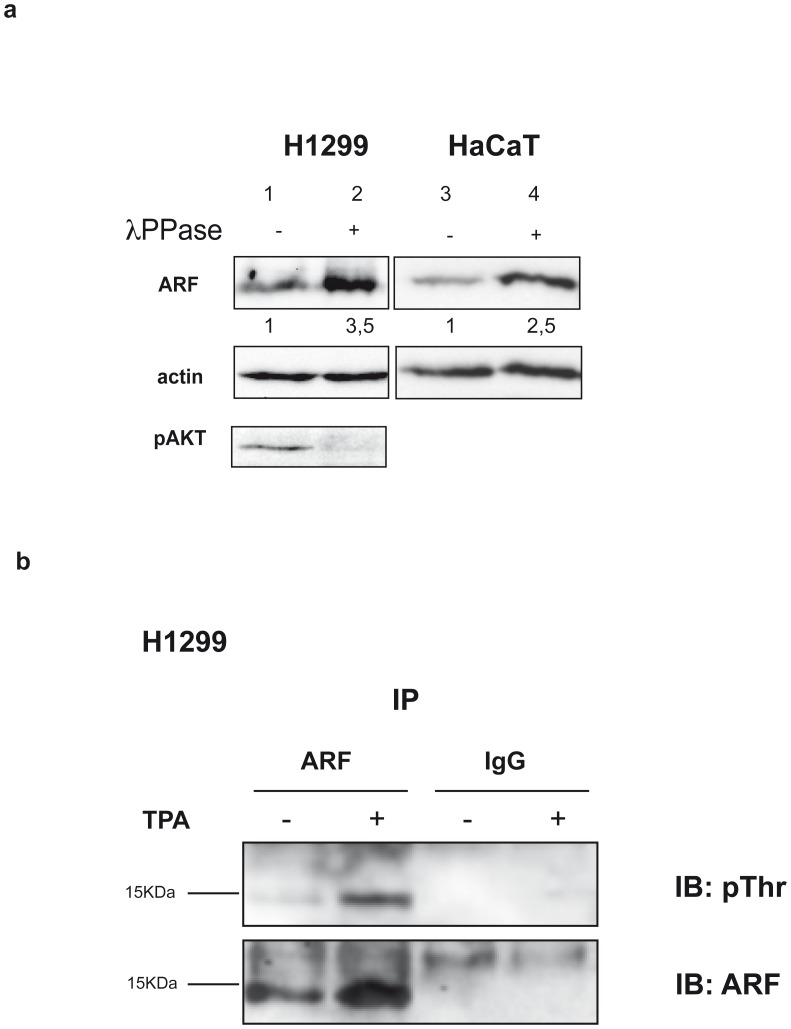
ARF phosphorylation assays. a) λ phosphatase assay: equal amount of HaCaT and H1299 cytoplasmic extracts were incubated (lane 2 and 4) or not (lane 1 and 3) with λ protein phosphatase (λ PPase), analysed by high resolving SDS-PAGE and blotted with anti ARF antibody. Actin is a loading control, while anti pAKT immunoblot is a reaction control. ARF band intensities, quantified as previously described, are also given. b) Immunoprecipitation: H1299 cells treated or not with TPA were immunoprecipitated with anti ARF antibody, subjected to SDS-page and blotted with anti pThr antibody (upper panel). The filter was stripped and incubated with anti ARF antibody (lower panel).

We next looked at the effect of TPA addition on the ARF protein levels both in nuclear and cytoplasmic compartments. As shown in Fig. 6b, following TPA treatment, only the cytoplasmic pool of ARF increased. Consistently, pPKC appeared upregulated in the cytoplasm. We thus decided to check the phosphorylation status of cytoplasmic ARF by a lambda phosphatase treatment (Fig. 7a). Equal amount of cytoplasmic extracts were incubated or not with lambda phosphatase and subjected to SDS-page and western blot with anti ARF antibody. The experiment clearly shows an increase of the ARF band in the treated sample (compare lane+with−) in both cell lines and using different ARF antisera (data not shown).

We next assessed ARF phosphorylation status in H1299 cells after TPA stimulation using a different approach. Cells were treated with TPA and protein extracts subjected to immunoprecipitation with anti ARF antibody followed by immunoblot with an antibody directed against phosphorylated threonine (pThr antibody). The experiment shows a 15 KDa band in untreated sample whose intensity increases following TPA stimulation (Fig. 7b upper panel). Identity of this band was confirmed by western blot with anti ARF antibodies (Fig. 7b lower panel).

### Mimicking ARF Phosphorylation Inhibits ARF Biological Activity

**Figure 8:**
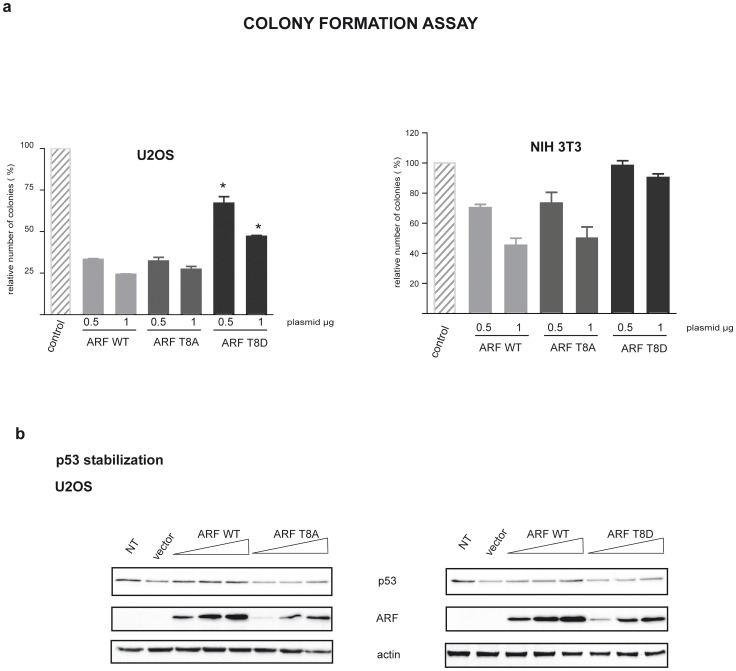
Growth suppression by wt and mutant ARF proteins. a) Graph indicating ARF and mutants ability to inhibit cell proliferation. U2OS and NIH cells were transfected with the indicated amounts of ARF and mutant expression vectors. After two weeks selection to isolate transfected colonies, cells were fixed and stained with crystal violet. The plot represents the percentage of colonies obtained with the indicated plasmids relative to that detected with the empty vector. Values represent the mean of four independent experiments. Standard deviation for each value is also given. Asterisks (*) indicate statistically significant differences (p<0.001) for the mutant samples compared to wt ARF. b) Western blot showing p53 stabilization following ARF (wt and mutant) transfection in U20S cells.

It has been repeatedly shown that ARF ectopic expression inhibits cell proliferation at least in cells expressing wt p53 [33], [35], [36]. Wild type and mutant ARF ability to block proliferation was tested by Colony formation efficiency assay (CFE assay) in U2OS cells where p53 pathway is intact. Results showed that, although less stable, T8A mutant was able to block cell proliferation with the same efficiency as the wt (Fig. 8a). Ectopic expression of the T8D mutant was instead less efficient (60% residual colonies after G418 selection in T8D versus 30% of wt and T8A, Fig. 8a). Similar results were obtained in the ARF null NIH 3T3 mouse cells harbouring wt p53 (Fig. 8a). Interestingly, western blot assays show that T8D ectopic expression is efficient in stabilizing p53 (Fig. 8b).

## Discussion

In this paper we report data showing that two known inducers of PKC activation, Ca^2+^ and TPA, both induce an increase of p14ARF protein level in immortalized and in tumor cell lines. The PKC family consists of at least ten serine/threonine kinases playing a central role in cell proliferation, differentiation, survival and death [37]. While classical isoforms need both calcium and the lipid molecules Diacylglycerol (DAG) or phorbol esters (TPA) for activation, novel kinases are calcium independent and lipid dependent, while atypical are both calcium and lipid independent. Our results suggest that the PKC isoform(s) involved might belong to the classical and/or novel subfamily. Accordingly, the involvement of PKCα in TPA-mediated stabilization of p14ARF has been reported [25]. PKCα, the major conventional Ca^2+^ responsive PKC in epidermis, is highly abundant in skin and is believed to play a key role in calcium induced keratinocyte differentiation [38], [39]. Remarkably, defective differentiation in skin cancer correlates with elevated PKCα activity. The positive correlation between tumor promoting effects of various phorbol esters with their ability to activate certain PKC isoforms, indicates that PKC activation is a critical step in skin tumor promotion [40]. Accumulating data show that PKC is linked to promotion and progression of several tumor types [41], [42], [43], [44] and a number of pharmacological PKC inhibitors are developed and currently used in clinical trial. However, PKCs play either as oncogenes or tumor suppressors in human cancer development [45] and, therefore, the combined effects of the various PKC isoforms can result in diverse, conflicting consequences depending on individual isozyme, cell-type and subcellular localization.

Our findings that ARF is regulated through the activation of PKC pathway led us to the hypothesis that ARF function might be regulated by phosphorylation. ARF contains numerous potential phosphorylation sites, and one study suggested that it could be a target of death associated protein kinase (DAPK) [46]. Moreover, the potential involvement of phosphorylation in controlling ARF activities has already been suggested by different experimental approaches [47], [48].

Our hypothesis is supported by several findings. First, sequence prediction analysis shows that three PKC consensus sequences are present within ARF protein sequence, and in vitro kinase assay shows that the protein can be specifically phosphorylated by PKC and dephosphorylated by PP1. In agreement, a protein complex consisting of activated PKC and ARF exists in cells, and we observed that immunoprecipitated ARF can be recognized by a pThr antibody in the H1299 cell line (Fig. 6b). Moreover, following TPA treatment, the amount of phosphorylated ARF species increase (Fig. 6b lower panel). Significantly, TPA-induced PKC activation results in the stabilization of cytoplasmic ARF.

Furthermore, we have functionally characterized the role of the conserved threonine 8 that lies within a PKC consensus sequence in ARF protein. While the T8A mutation does not affect ARF localization, the phospho-mimetic T8D mutation dramatically reduces ARF ability to be exclusively localized in the nucleoli inducing the protein to accumulate in the cytoplasm in a significative fraction of cells. The observation that λ phosphatase treatment results in a clear thickening of the ARF electrophoretic band, suggests that the cytoplasmic protein is indeed phosphorylated. On the basis of these results we speculate that multiple phosphorylated ARF species are present in the cytoplasm, and, after phosphatase treatment, converge to the 15Kd band, increasing its intensity. Accordingly, the MBPT8A ARF mutant is still a PKC substrate in vitro (Fig. 2c), suggesting that multiple sites could be phosphorylated within the endogenous protein, as indicated by in silico analysis. Finally, while the T8A mutant retains the ability to inhibit proliferation as the wt protein, the phosphomimetic mutant T8D is severely impaired in the control of cell proliferation. Interestingly, both MDM2 binding and p53 stabilization appear to be unaffected by the T8D mutation. These results are in agreement with the notion that ARF dependent p53 activation takes place in the nucleus [47], [49], and suggest that the partial loss of function observed with the T8D mutant could be ascribed to p53 independent ARF activities specifically impaired by the T8D mutation. Our data also suggest that, at least in a p53 plus cell environment, the unphosphorylated protein is active in blocking cell proliferation.

Analysis of protein turnover shows that the T to A substitution strongly reduces protein half-life from six to two hours. Despite its short half-life, T8A retains both nucleolar localization and control of cell progression abilities. Given that it is expressed at very low levels respect to the wt protein, the experiments indicate that few active ARF molecules can guarantee an efficient control of cell proliferation [50], [51].

Altogether our data suggest that, following activation of PKC, ARF protein is phosphorylated and accumulates in the cytoplasm where it is unable to efficiently control cell proliferation. Further experiments are needed to precisely identify the molecular pathway, the potential involvement of additional kinase/s and the ARF residues directly involved in this mechanism.

In conclusion we suggest that in a tumor environment, where PKC is activated, ARF levels increase but its growth inhibitory function is impaired. Interestingly, despite its role in growth suppression, ARF is overexpressed in a significant fraction of human tumors [52]. These observations, together with our findings underscore the need for a careful re-evaluation of the status and role of p14ARF in human cancer.

Our observations uncover a novel mechanism through which cancer cells might escape ARF surveillance during cancer progression. A deeper understanding of the PKC dependent ARF functions may provide previously unknown and critical therapeutic targets for cancer treatment.

## Materials and Methods

### Cell Culture, Cloning, Protein Expression and Reagents

The human epithelial cell line HaCaT was obtained from Dr. Antonio Costanzo, Department of Dermatology, University of Rome Tor Vergata, 00133 Rome, Italy. All other cell lines (U2OS and H1299) were purchased from the American Type Culture Collection (ATTC). Cells were grown in Dulbecco’s modified Eagle medium supplemented with 10% fetal bovine serum (Euroclone, Life Science) at 37°C in a humidified atmosphere of 5% (v/v) CO2 in air. Cells were transfected using LipofectAMINE 2000 reagent (Invitrogen). For CFE analysis U2OS were trasfected with calcium phosphate precipitation as previously described [53].

The T8 amino acid changes were inserted into human p14ARF by site-directed mutagenesis using a wild-type p14ARF cDNA template. Standard polymerase chain (PCR) reaction conditions were used with each set of primers containing the desired mutations, and all products were verified by sequencing before subcloning into pMal, pcDNA3.1 and pEGFP vectors.

Antibodies used in this study: anti PARP-1 and anti-phospho-PKC (pan) antibody (Beta IISer660) (from Cell Signaling Technologies 9542, Boston, MA, USA); anti RACK-1 B3, anti ARF C-18, anti actin I-19, Anti HistoneH1 N-16, anti p53 DO-1, anti MDM2 SMP-14, (from Santa Cruz); anti ARF 4C6/4 (Abcam); anti ARF Ab2 14PO2 (Neomarkers); 6XHIS monoclonal antibody (Clontech); anti myc antibody 06549 (Upstate); anti X-press monoclonal antibody (Invitrogen); anti pThreonine Q7 (Qiagen). Western blot (WB) analysis was performed as previously described [29]. The molecular weight marker used for SDS page is the PageRuler™ Prestained Protein Ladder (Fermentas and Pierce). Extracts were blotted onto PVDF Immobilon-P transfer membrane (Millipore cat. NO. IPVH00010). Proteins were visualized with an enhanced chemiluminescence detection system (Amersham ECLplus ™) and images were taken with ChemiDoc XRS System (Bio-Rad Laboratories) and analysed with the QuantityONE software except when differently indicated.

Reagents used in this study: TPA was purchased from Applichem, Cycloheximide and Bisindolylmaleimide from Calbiochem. TPA and cycloheximide were dissolved in DMSO and used at 10µM and 80 µg per ml respectively. Bisindolylmaleimide (Bim) was used at 5µM final concentration. ARF band intensities following various treatments (Fig. 1–6–7) were quantified, normalized to actin (or to histone H1 in case of nuclear extracts in fig. 6) and reported as fold increase or decrease compared to untreated sample. Each value represents the mean of three independent experiments ± standard deviations.

### Protein Turnover Analysis

For half-life studies, transfected U20S were replated onto 6-well dishes, allowed to adhere overnight, and treated with cycloheximide or left untreated (0 hour control) for various time points. Cells were harvested and p14ARF protein levels examined by WB analyses of whole cell lysates with anti ARF antibody (C-18, Santa Cruz) and actin as loading control. Band intensities at the different time points were quantified by Image J Software, normalized to actin and reported in graph as percentage of total protein (protein a t0). Each profile represents the mean of three independent experiments. Standard deviations are also shown.

### Subcellular Localization Assay

U2OS cells (5.0×10^4^) were plated in 35 mm dish on micro cover glasses (BDH) and transfected with 0.5µg of plasmid expressing wt or mutant ARF. At 24 hrs after transfection with the indicated vectors, cells were washed with cold phosphate-buffered saline (PBS) and fixed with 4% paraformaldehyde (Sigma-Aldrich, Germany) for 15 min at RT. Cells were permeabilized with ice cold 0.5% Triton X-100 for 5 min and then washed with PBS. Subcellular localization were determined by using the monoclonal antibody against 6XHis, 1h a RT, followed by incubation with a Cy3-conjugated anti-mouse antibody (ImmunoResearch Laboratory) at 37°C for 30 min. After PBS/0.05% Tween washing, the cells were incubated with DAPI (Sigma-Aldrich, Germany) for 3 min and washed with PBS/0.05% Tween. Coverslip were mounted with Vectashield (VectorLab) and examined under a fluorescence microscope (Nikon). For each transfection point, subcellular localizations were analysed in 100–150 cells and results plotted in graph. Histograms (% of transfected cell ± S.D.) represent the mean of at least three independent transfection experiments. Statistical analysis of variance (Anova plus Bonferroni post test) was performed using GraphPad Prism 5.0 software.

Subcellular fractionation of H1299 and HaCaT cells was carried out as by Colucci-D’Amato et al., 2000 [54] with minor modifications. In order to analyse ARF protein distribution in the nuclear and cytoplasmic compartments, same number of cells and nuclei were processed as described. Cells were harvested, washed in ice cold PBS, resuspended in hypotonic buffer (Hepes 10mM, KCl 60mM, DTT 1mM plus protease inhibitors) and incubated on ice for 20 minutes. Following addition of NP-40 (0.2% final concentration) cells were further incubated 1 minute on ice and spun at 600g for 5 minutes. Supernatants were subsequently centrifuged at 13000 rpm for 15 min to pellet unbroken nuclei and organelles to obtain clear cytoplasmic lysates. Nuclei were briefly washed in hypotonic buffer to eliminate traces of detergent, purified on sucrose cushion, counted and lysed in RIPA buffer. To verify the purity of the subcellular fractionation, anti-PARP1, anti Histone H1, anti-rack1 and anti actin antibodies have been used in WB.

### Proliferation Assay

For the CFE assay (colony formation efficiency assay) U2OS (1×10^5^ cells per well) cells were seeded into 6-well multiplates, transfected with 2 µg of total plasmid DNA using the standard calcium phosphate method and processed as previously described [53]. Graphs (% of remaining colonies ± S.D.) represent the mean of at least four experiments. Statistical analysis of variance was performed as previously described.

### p53 Stabilization Assays

Stabilization of p53 was performed in U2OS transfected with wt, T8A and T8D mutant plasmid constructs. Protein levels were analysed 24h after transfection with anti ARF (C-18), anti p53 (DO-1) and anti actin antibody as loading control.

### Phosphorylation Assays

The MBP::ARF fusion proteins (both wt and mutants) were obtained as previously described [53] and subjected to an in vitro phosphorylation assay using a commercially available kit following manufacturer instructions (Stratagene). PP1 dephosphorylation assay was performed with the catalytic PP1 subunit (Promega) following manufacturer instructions.

Lambda-phosphatase (λ-PPase; New England Biolabs) treatment was performed as previously described [55]. Immunoprecipitation following TPA treatment: 4 mg of total cellular extracts of H1299 cells treated or not with TPA (10µM for 2 min) were lysed in IP buffer (Tris-HCl pH7 10mM, NaCl 150 mM, SDS 0.1%, Triton-X100 1%, EDTA 1mM, EGTA 0,5mM) plus protein phosphatase inhibitors. Cleared lysates were incubated with 3µg of anti ARF (Ab2 14PO2, Neomarkers) ON. After washing immunocomplexes were subjected to SDS-page on a 13% gel followed by incubation with anti pThreonine (1∶100 dilution in 3%BSA) followed by chemioluminesce (Pierce). The filter was then stripped and incubated with anti ARF antibody (C-18) in order to visualize total immunoprecipitated ARF protein.

### Co-Immunoprecipitation

ARF/pPKC co-immunoprecipitation assay were performed with 2 mg of total cellular extracts. Cells were harvested, lysed with co-ip buffer (Tris-HCl pH7 10mM, NaCl 150 mM, NP40 0.5%, glycerol 10%, EDTA 0.5mM) and incubated ON with anti ARF antibody (C-18 Santa Cruz). After washing immunocomplexes were subjected to WB and analysis with anti pPKC pan antibody (Beta IISer660, Cell Signalling), anti ARF (Ab2 14PO2, Neomarkers) antibody and anti MDM2 antibody.

ARF (wild type and mutants)/MDM2 or myc tagged p32 co-immunoprecipitation assays were performed with 1 mg of total cellular extracts. Twenty-four hours following transfection cells were harvested and processed as described. Ip were carried out with anti X-press antibody and immunoprecipitated samples analysed by WB with anti MDM2, anti myc and anti ARF antibodies.
