# Correction: Mimicking p14ARF Phosphorylation Influences Its Ability to Restrain Cell Proliferation

**DOI:** 10.1371/annotation/77e996e1-a18e-47a6-918b-801932929b28

**Published:** 2013-10-30

**Authors:** Maria Vivo, Michela Ranieri, Federica Sansone, Cristina Santoriello, Raffaele A. Calogero, Viola Calabrò, Alessandra Pollice, Girolama La Mantia

The image reported in Figure 3A, IP anti X-press, in the lower right hand panel (corresponding to the immunoprecipitated T8D protein) is incorrect. It corresponds to the flipped version of the Western blot data shown in the lower middle panel (corresponding to the immunoprecipitated T8A protein). The authors apologize for this error. Please refer to the corrected Figure 3 where the right T8D bands have has been included and to the raw data provided.

Figure 3: 

**Figure:**
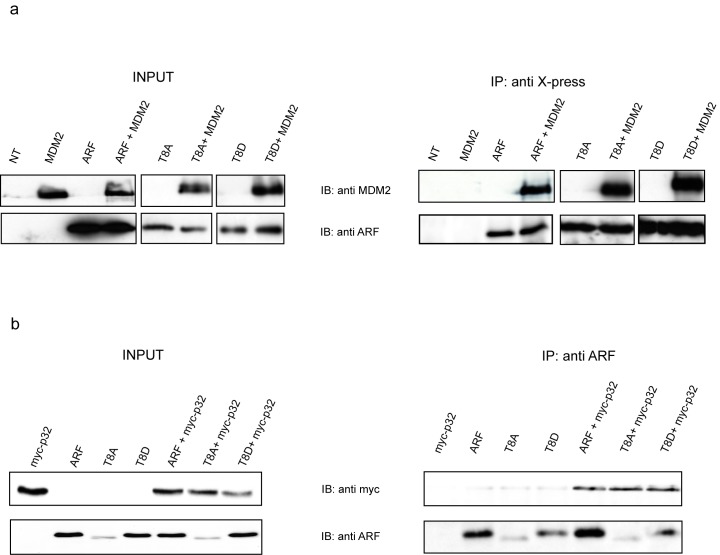


Raw Data: http://www.plosone.org/corrections/pone.0053631.g003.1.cn.tif

